# Corneal neovascularization during experimental fungal keratitis

**Published:** 2009-09-29

**Authors:** Xiaoyong Yuan, Kirk R. Wilhelmus

**Affiliations:** Sid W. Richardson Ocular Microbiology Laboratory, Cullen Eye Institute, Department of Ophthalmology, Baylor College of Medicine, Houston, TX

## Abstract

**Purpose:**

To investigate the development of corneal neovascularization, the corneal expression of vascular endothelial growth factor (VEGF), and the antiangiogenic effects of a VEGF-inhibitory antibody during experimental keratomycosis.

**Methods:**

Scarified corneas of BALB/c mice were topically inoculated with *Candida* *albicans* and monitored daily for corneal neovascularization. A murine gene microarray compared infected corneas to controls 1 day after inoculation. Real-time reverse transcriptase polymerase chain reaction (RT-PCR) determined levels of genes encoding VEGF-A, VEGF-B, VEGF-C, and VEGF-D and placental growth factor in infected, mock-inoculated, and normal corneas. Immunostaining localized VEGF-A in corneal sections. An anti-VEGF-A antibody that binds to murine VEGF was evaluated for effects on corneal neovascularization and fungal recovery.

**Results:**

Eyes with *C. albicans* keratitis manifested limbal capillary budding on the second postinoculation day, and intrastromal neovascular tufts subsequently grew at a mean rate of 250±80 μm/day. One day after the onset of *C. albicans* keratitis, *VEGF-A* was upregulated 12.5 fold (p=0.01) by microarray and 8.8 fold (p=0.004) by real-time RT-PCR, followed by a measured decline toward baseline over one week. VEGF-A was present in the epithelium and stroma of infected corneas. Scarification alone did not alter VEGF expression compared to the normal cornea. Anti-VEGF-A antibody significantly (p<0.01) decreased the formation of new corneal blood vessels during experimental keratomycosis without adversely affecting the fungal load of *C. albicans* keratitis.

**Conclusions:**

Untreated *C. albicans* keratitis induces VEGF-A and leads to progressive corneal neovascularization that is preventable by a VEGF-blocking antibody.

## Introduction

New vessels form and grow in the normally avascular cornea when the homeostatic balance is upset by infection and inflammation [1,2]. Angiogenic factors that promote ocular neovascularization include the vascular endothelial growth factor (VEGF) family [3]. As neovascularization may worsen visual prognosis, anti-VEGF inhibitors offer the possibility of controlling sight-threatening neovascular disorders of the eye [4,5].

Corneal neovascularization complicates *Candida albicans* keratitis [6], but the molecular pathogenesis of angiogenesis during fungal keratitis has not yet been studied. We used a murine model of posttraumatic *C. albicans* keratitis to determine the corneal VEGF profile during the onset and progression of fungal keratitis. We also studied the effect of VEGF-blocking treatment during experimental *C. albicans* keratitis. Because a humanized anti-VEGF antibody such as bevacizumab weakly interacts with murine VEGF-A [7], we used a cross-reactive monoclonal antibody constructed with a murine immunoglobulin constant domain to block the interaction of murine VEGF with ocular VEGF receptors [8]. Before studying the efficacy of subconjunctival or topical application in the mouse model, we used a proof-of-principle approach by administering anti-VEGF antibody systemically at a dosage capable of inhibiting corneal neovasularization [9].

## Methods

### Fungi

*C. albicans* strain SC5314 is a clinical isolate capable of producing experimental keratomycosis [10]. Yeasts were cultured on Sabouraud dextrose agar (Difco, Detroit, MI) for 3 days at 25 °C. Colonies were harvested and diluted in sterile phosphate-buffered saline (PBS) to yield 2×10^5^ colony-forming units (CFU)/μl based on an optical density (OD) at 600 (OD_600_) nm with a conversion factor of 1 OD_600_ unit equal to 3×10^7^ CFU/ml.

### Animals

Animals were treated in accordance with the ARVO Statement for the Use of Animals in Ophthalmic and Vision Research under protocols approved by the Baylor College of Medicine Institutional Animal Care and Use Committee. Female BALB/c mice and C57BL/6J mice 6 to 8 weeks of age (Harlan Sprague-Dawley, Houston, TX) were anesthetized intraperitoneally with ketamine, xylazine, and acepromazine, and the corneas of right eyes were superficially scarified [10]. A 5 μl inoculum of either *C. albicans* (1×10^6^ CFU) or sterilized PBS buffer was topically applied to eyes of infected and control groups, respectively. Mice were monitored daily for 7 days post inoculation (p.i.) using a dissecting microscope to categorize corneal inflammation [10]. The amount of corneal neovascularization was assessed by a scoring system modified from a semiquantitative method [11] that assigned grades of 0 to 4 for number, density, and length of visible corneal blood vessels (Table 1). Corneal photographs with the eye positioned in lateral profile were captured with a Zeiss photo slit-lamp and Nikon digital camera. Imported images were converted to linear gray-scale equivalents using SigmaScan image-analysis software (Systat, Richmond, CA), and the limbal arcade and neovascular network were manually delineated based on adjacent pixel values for edge detection [12].

**Table 1:** Criteria used in grading severity of corneal neovascularization.

**Score**	**Number of quadrants with corneal neovascularization**	**Number of corneal vessels/quadrant**	**Proportionate length of longest corneal vessels between limbus and corneal center**
1	1	1-5	0.05-0.25
2	2	6-10	0.26-0.50
3	3	11-15	0.51-0.75
4	4	>15	>0.75

### RNA extraction

Mice were sacrificed 1, 3, and 7 days p.i., and eyes were enucleated for analysis. Corneas were dissected, and surrounding conjunctiva and uvea were removed. Three cornea pools (5 corneas/pool) were prepared from *C. albicans-*infected and mock control groups at days 1, 3, and 7 p.i. and from untreated normal mouse corneas, respectively. RNA was extracted by a previously reported procedure [13]. Total RNA was isolated with RNeasy MicroKit columns (Qiagen, Valencia, CA). Samples were treated with DNase (Qiagen) to exclude DNA contamination and stored at -80 °C until use.

### Gene microarray

Microarray was performed by the Microarray Core Facility of Baylor College of Medicine as reported [13]. After checking RNA samples for quality assurance, Genechip (Affymetrix, Santa Clara, CA) microarray protocols were applied to qualified samples of 3 five-cornea pools from *C. albicans-*infected and mock control groups for two cycles of amplification. Images and quality control metrics were recorded using Affymetrix GCOS software version 1.4, and raw signal intensity data were adjusted and analyzed with BioConductor software. The criterion for significance of differentially regulated genes was >2 fold change with adjusted p<0.05.

### Quantitative polymerase chain reaction

Total RNA isolated from 3 pools (5 corneas/pool) at 1, 3, or 7 days p.i. respectively was quantified by absorbance at OD_260_. The first-strand cDNA was synthesized from 0.4 μg of total RNA with Ready-To-Go You-Prime First-Strand Beads (GE Healthcare, Princeton, NJ) and random hexamers (Applied Biosystems, Foster City, CA). Real-time PCR was performed using TaqMan Gene Expression Master Mix and Assays (Applied Biosystems). Primers specific for *VEGF-A*, *VEGF-B*, *VEGF-C*, *VEGF-D*, and placental growth factor (*PlGF*) transcripts (Applied Biosystems) were used to quantify gene expression levels. The threshold cycle (C_T_) for each target mRNA was normalized to glyceraldehyde-3-phosphate dehydrogenase (*GAPDH*) mRNA and averaged. Three five-cornea pools were processed for each group. Two-group comparisons were done using the Student *t*-test, and three-group comparisons used one-way analysis of variance (ANOVA). For longitudinal analysis of VEGF transcriptional levels, mean results were compared with ANOVA using a pairwise multiple comparison procedure. A p<0.05 was considered statistically significant.

### Immunofluorescence

Three eyes from each group obtained 1 day p.i. were embedded in OCT compound (Sakura Finetek, Torrance, CA), snap-frozen in liquid nitrogen, and sectioned at 15 μm thickness. Sections were thawed, dehydrated, and fixed in 2% paraformaldehyde then blocked with 10% normal donkey serum (Jackson ImmunoResearch Laboratories, Philadelphia, PA). Immunofluorescent staining was performed as reported [14]. Polyclonal goat antibody to the NH_2_-terminus of mouse VEGF-A (sc-1836; Santa Cruz Biotechnology, Santa Cruz, CA) was diluted 1:100, and applied to the blocked sections that were incubated overnight at 4 °C. Secondary Alexa-Fluor 488-conjugated donkey anti-goat antibody (Invitrogen, Carlsbad, CA) was applied to sections that were incubated in a dark chamber for 1 h and counterstained with propidium iodine (Invitrogen) in Gel/Mount (Biomeda, Foster City, CA). Sections were observed with a laser-scanning confocal microscope (LSM 510; Zeiss, Thornwood, NY) with 488- and 543-nm excitation and emission filters. Images were acquired with a 40× oil-immersion objective and processed using Zeiss LSM-PC software.

### Anti-VEGF treatment

A buffered formulation of a phage library-derived anti-VEGF antibody, B20-4.1.1 [8], that blocks both human and mouse VEGF-A (Genentech, South San Francisco, CA) was diluted in PBS to a dosage of 5 mg/kg. Five mice were allocated to treated and control groups, respectively, and each animal received 200 μl of either B20-4.1.1 or PBS, injected intraperitoneally 5 days, 3 days, and 1 day before corneal scarification and topical inoculation of *C. albicans* 1×10^6^ CFU/5 μl. Eyes were observed daily with a dissecting microscope to grade the severity of keratitis and the extent of corneal neovascularization.

### Quantitative fungal culture

Each of ten additional BALB/c mice or C57BL/6J mice were treated intraperitoneally with either B20-4.1.1 or PBS on 5 days, 3 days, and 1 day before fungal inoculation and then sacrificed one day p.i. for quantitative fungal recovery from excised corneas by previously reported method with some modifications [10]. Excised corneas were homogenized by a frosted-glass grinder with 500 μl PBS, and the homogenate aliquot was 10 fold diluted and cultured on Sabouraud dextrose agar for 4 days at 25 °C. Visible colonies were counted and compared in B20-4.1.1- and PBS-treated groups.

## Results

### Experimental fungal keratitis

**Figure 1:**
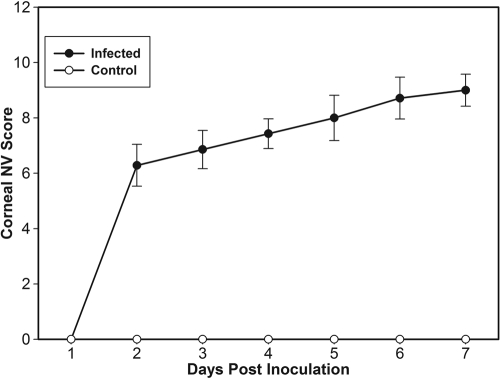
Severity evaluation of corneal neovascularization during *C. albicans* infection. Relative corneal neovascularization (NV) was compared in eyes with *C. albicans* keratitis (infected) and in scarified, mock-inoculated eyes (control). Each point represents the mean neovascularization score (±SD) of 5 eyes at each day following topical inoculation.

**Figure 2:**
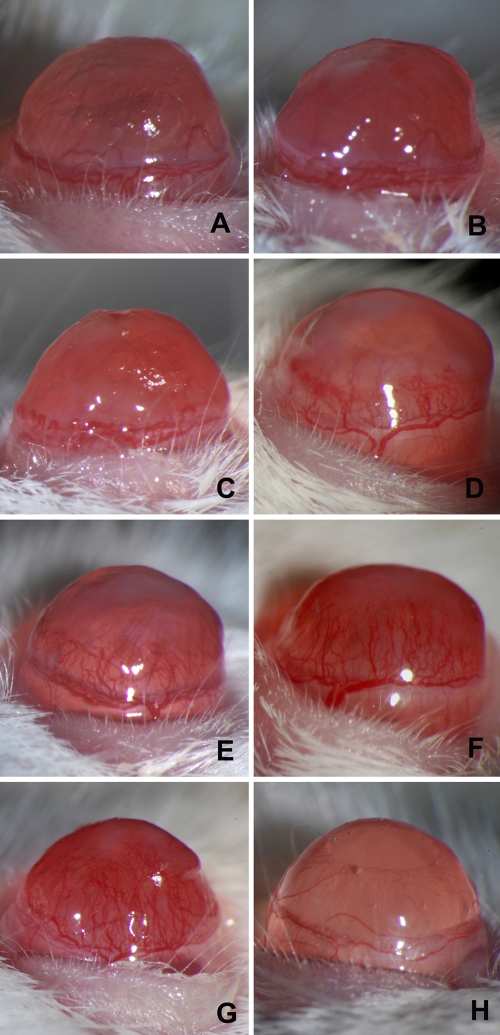
Corneal neovascularization during 7 days of follow up during murine *C. albicans* infection. Daily progression over one week of corneal neovascularization in mouse eyes with *C. albicans* keratitis (panels **A** to **G** from day 1 p.i. to day 7 p.i., respectively). No abnormal corneal blood vessels occurred in the mock-inoculated control (**H**).

All corneas inoculated with *C. albicans* developed signs of inflammation and neovascularization. Congestion of the limbal pericorneal plexus began 1 day p.i., and capillary budding of limbal vessels occurred 1 to 2 days later (Figure 1). Corneal vessels continued to extend toward the area of inflammation at the rate of 0.25±0.08 mm/day and reached the central cornea on days 6 to 7 (Figure 2). Neither corneal inflammation nor neovascularization occurred among mock controls or normal mice.

### VEGF gene expression profile

Gene arrays of *C. albicans*-infected corneas and mock-inoculated control corneas were compared for VEGF expression (Table 2). Ratios of expression levels at 1 day p.i. showed that *VEGF-A* was upregulated an average of 12.5 fold (p=0.01). *VEGF-B* was downregulated -2.8 fold (p=0.002). Neither *VEGF-C*, *VEGF-D*, nor *PlGF* differed significantly between infected eyes and controls. Transcript levels detected by quantitative real-time RT-PCR were consistent with microarray findings (Table 2). Table 3 shows the average real-time RT-PCR C_T_ values among the three groups. Compared to mock-inoculated controls, *VEGF-A* transcript level was upregulated 8.1 fold (p=0.004) at day 1 p.i., followed by 5.4 fold (p=0.01) at day 3 p.i. and 2.5 fold (p=0.23) at day 7 p.i. Other VEGF family members did not increase significantly during follow up. *VEGF-B* was downregulated -2.5 fold (p=0.04), and *VEGF-D* was downregulated -3.9 fold (p=0.0004) on day 1 p.i. Compared to normal eyes, mock-inoculated controls were not significantly different in *VEGF-A*, *VEGF-B*, *VEGF-C*, *VEGF-D*, or *PlGF* expression levels.

**Table 2:** Microarray analysis and real-time RT-PCR confirmation of *VEGF* expression ratios comparing *C. albicans* keratitis to mock-infected controls.

**Molecule**	**GenBank accession number**	**Mean signal intensity ratio±SD by microarray**	p*	**Pooled mean signal intensity ratio±SD by microarray**	p**	**Mean fold change±SD by real-time RT-PCR**	**p#**
*VEGF-A*	AC127690	11.5±2.9	0.002	12.5±4.4	0.65	8.79±4.46	0.27
	AB086118	13.4±6.1	0.01				
*VEGF-B*	AK148188	-2.8±0.2	0.002	-2.8±0.2	1.0	-2.87±1.69	0.95
*VEGF-C*	AC120547	1.4±0.2	0.53	1.3±0.2	0.36	-0.90±2.19	0.008
	AC120547	1.3±0.1	0.33				
	AC163012	1.2±0.1	0.22				
*VEGF-D*	BC030037	-1.4±0.3	0.09	-1.9±1.6	0.48	-3.91±0.67	0.066
	BC062809	-1.5±2.3	0.23				
	BC080770	-2.9±1.5	0.022				
*PlGF*	AK042891	1.8±0.6	0.13	1.8±0.6	1.0	0.50±1.68	0.28

The asterisk indicates statistical comparison of microarray signals between infected and control groups. The double asterisk indicates statistical comparison of gene expression results among genomic probes. The sharp (hash mark) indicates statistical comparison of pooled microarray and real-time RT-PCR results. Abbreviations in the table are: *PlGF*, placental growth factor; RT-PCR, reverse transcription-polymerase chain reaction; SD, standard deviation; *VEGF*, vascular endothelial growth factor.

**Table 3:** Quantitative gene expression levels.

**Gene**	**Normal cornea**	**Mock-infected cornea**			**Infected cornea**		
		**Day 1**	**Day 3**	**Day 7**	**Day 1**	**Day 3**	**Day 7**
*VEGF-A*	7.20±0.52	7.73±0.36	7.99±0.30	6.84±0.97	4.71±0.80	5.56±0.91	5.54±1.26
*VEGF-B*	5.72±0.38	6.64±0.17	6.59±0.51	5.83±0.95	7.98±0.78	8.05±0.77	6.38±1.24
*VEGF-C*	9.80±0.07	9.92±0.21	10.97±0.65	9.49±2.10	10.34±0.92	10.69±0.28	8.70±1.61
*VEGF-D*	8.49±0.58	7.60±0.12	7.85±0.19	7.90±0.07	9.55±0.28	8.54±0.50	7.36±0.19
*PlGF*	8.50±0.98	8.70±0.14	8.14±0.33	8.18±0.57	8.52±0.76	8.60±0.51	7.64±0.60

Mean threshold cycle number ±standard deviation normalized to *GAPDH* by real-time RT-PCR.

### VEGF-A protein expression pattern

**Figure 3:**
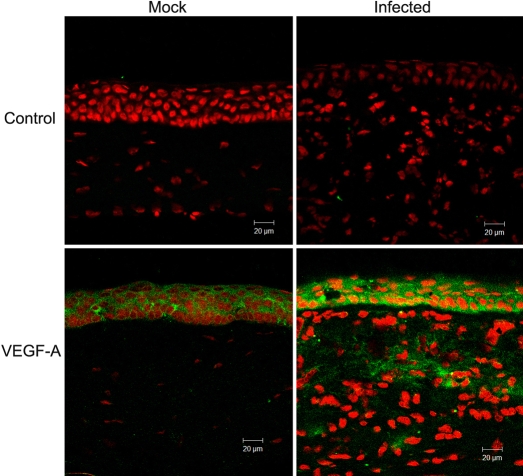
Molecular expression patterns in situ in corneas with *C. albicans* infection. VEGF-A expression was compared between corneas with *C. albicans* keratitis (Infected) and mock-inoculated controls (Mock). Negative controls lacked primary antibody (Control”. Corneal sections were processed with anti-VEGF-A monoclonal antibodies (VEGF-A). Immunofluorescence showed VEGF-A in the healed epithelium of scarified corneas and within the epithelium and stroma one day after the onset of experimental *C. albicans* keratitis. Original magnification, 10×. Scale bar, 20 μm.

The in situ pattern determined by immunofluorescent staining showed moderate epithelial staining for VEGF-A among normal eyes and scarified corneas. At 1 day p.i., corneas from infected eyes had increased staining for VEGF-A throughout epithelial and stromal layers (Figure 3).

### VEGF kinetic analysis

**Figure 4:**
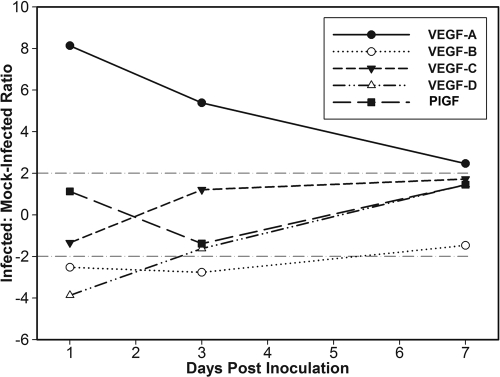
Relative *VEGF* gene expression during *C. albicans* keratitis. Differential gene expression ratios of vascular endothelial growth factors were determined by real-time RT-PCR throughout the first week. Gene expression levels in corneas with posttraumatic *C. albicans* keratitis were compared to levels in scarified, mock-inoculated control corneas. Horizontal dashed lines indicate 2 fold threshold expression levels.

Real-time RT-PCR on total RNA extracted from groups of five-cornea pools showed differences between *C. albicans* keratitis and scarified controls at 1, 3, and 7 days p.i. (Table 3). In infected corneas, *VEGF-A* transcripts were upregulated on day 1 p.i. then declined toward baseline levels but remained significantly increased at 3 days p.i. (Figure 4). *VEGF-B* and *VEGF-D* were slightly downregulated at day 1 p.i., and *VEGF-B* remained relatively downregulated in infected corneas on day 3 p.i. By day 7 p.i., *VEGF-B* and *VEGF-D* levels in experimental corneas were similar to controls and normal eyes. *VEGF-C* and *PlGF* remained unchanged in infected corneas compared to controls.

### Treatment effects of anti-VEGF antibody

**Figure 5:**
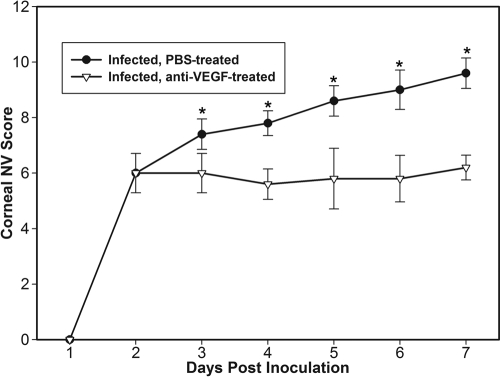
Effect of anti-VEGF pretreatment on corneal neovascularization during fungal keratitis. Relative severity of corneal neovascularization (NV) during 7 days of follow up in *C. albicans* keratitis in mice treated with intraperitoneal VEGF-blocking antibody B20-4.1.1 (anti-VEGF) compared to controls receiving phosphate-buffered saline (PBS). Each point reprsents the mean neovascularization score (±SD) of 5 eyes at each day following topical inoculation. Asterisks indicate time points having a statistically significant (p<0.01) difference.

**Figure 6:**
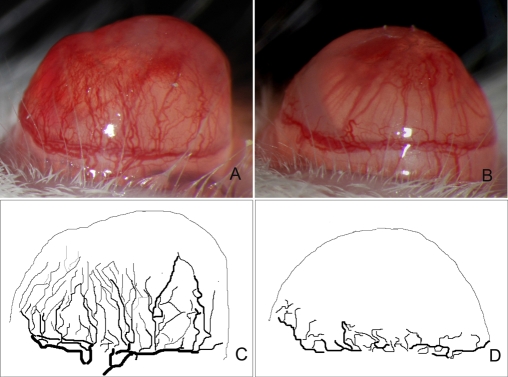
Inhibition of corneal neovascularization by anti-VEGF antibody. Comparison of corneal neovascularization in control (**A** and **C**) and anti-VEGF-treated mice (**B** and **D**) at 7 days p.i. **A**: *C. albicans* keratitis results in multiple blood vessels arising from the limbal arcade and extending toward the central cornea. **B**: Treatment with VEGF-blocking antibody results in fewer and shorter corneal blood vessels that remain limited to the peripheral cornea. **C**: Image analysis of corneal blood vessels in a PBS-treated mouse. **D**: Image analysis of corneal blood vessels in anti-VEGF-treated mouse, ignoring underlying radial iris vessels that had a slightly larger caliber but lacked visible iris neovascularization.

Compared with PBS-injected animals, corneal neovascularization in anti-VEGF-treated mice was significantly reduced, and this effect persisted until 15 days p.i. when observations ceased. An inhibitory effect was apparent by 3 days p.i. (p=0.008), and treated animals continued to have less corneal neovascularization on each subsequent day (p<0.001) (Figure 5). At 7 days p.i., the average vascularization score of 6.2±0.5 in treated mice remained significantly lower (p=0.0002) than the average score of 9.6±0.6 in controls. Image analysis confirmed that fewer blood vessels were present in the peripheral cornea in anti-VEGF-treated mice compared to PBS-treated mice (Figure 6). Severity scores of corneal inflammation were not significantly different between treatment and control groups at any day during one week of observation (p>0.05), although slightly more prominent iris vessels were noted in the anti-VEGF-treated group. Cultures from excised BALB/c mice corneas at 1 day p.i. showed no significant difference (p=0.63) in the mean±SD number of viable fungi recovered from PBS-treated mice (28,750±37,979 CFU/cornea) compared to those treated with anti-VEGF antibody (20,110±9,550 CFU/cornea). Similarly, for C57BL/6J mice, no significant difference (p=0.62) was found for the recovery cultures between PBS-treated mice (20,100±3,719 CFU/cornea) and anti-VEGF antibody-treated mice (21,750±6,072 CFU/cornea).

## Discussion

Fungal infection of the cornea provokes stromal inflammation and neovascularization [6]. The innate immune response triggers the production of inflammatory mediators soon after fungal adherence and invasion [13,14]. Corneal neovascularization occurs in response to angiogenic mediators released by leukocytes and corneal cells [15,16].

We confirmed that *C. albicans* keratitis incites corneal neovascularization, with angiogenesis beginning sooner in the infected mouse eye than in the rabbit model [6]. New blood vessels bud from the murine pericorneal plexus within 2 to 3 days after the onset of corneal infection and inflammation. Progressive neovascular extension toward the central cornea contributes to corneal opacification during fungal keratitis.

VEGF-A has a pivotal role in inflammatory neovascularization [17]. During experimental keratitis VEGF-A is increased throughout the corneal epithelium and stroma [18,19] and is extensively expressed in the inflamed, vascularized cornea [20,21]. Our results with comparative genomics and immunopathology confirmed that VEGF-A is present in the corneal epithelium [22] and increases throughout the cornea soon after the onset of experimental fungal keratitis. VEGF-A expression is closely followed by limbal vascular sprouting into the peripheral conea.

The brisk increase of VEGF-A during *C. albicans* keratitis parallels VEGF production during experimental *Pseudomonas aeruginosa* keratitis [23-25]. Our findings are also consistent with studies showing that systemic infection by *C. albicans* produces neovascularization adjacent to fungal microabscesses [26]. VEGF expression increases upon exposure to virulent *C. albicans* [27] and triggers local cytokine production. The upsurge in interleukins and other local cytokines that occurs at the onset of *C. albicans* keratitis [13] leads to recruitment of leukocytes that contribute to VEGF production [23,28].

Our findings indicate that VEGF mediates corneal neovascularization during keratomycosis. VEGF-deficient transgenic mice could not be used to confirm this inference because VEGF is essential for embryogenesis and survival [29,30]. VEGF-A appears closely involved with the neovascular process during fungal keratitis. Our previous studies also suggest that proinflammatory matrix metalloproteinases (MMPs) may have a role in corneal neovascularization. MMP-9 increases during fungal keratitis [14] and is capable of promoting angiogenesis during stromal degradation [31]. Fungal keratitis consists of a coordinated interplay of inflammatory and neovascular mediators that offer possible targets for intervention.

Inhibitors of VEGF-A might have a therapeutic role in the management of corneal disease. Corticosteroids and other anti-inflammatory drugs reduce vascular ingrowth during fungal keratitis [6,32] but can potentiate fungal replication [33]. Anti-VEGF antibodies provide a specific intervention to slow the onset and progression of corneal neovascularization.

Bevacizumab inhibits inflammatory corneal neovascularization in experimental animal models [9,34-37]. Because this humanized antibody has weak activity against murine VEGF [7], we used a monoclonal antibody that blocks murine VEGF-A activity and examined its effects on experimental fungal keratitis [8,38]. Systemic anti-VEGF administration effectively inhibited corneal angiogenesis that occurs during *C. albicans* keratomycosis but did not adversely alter corneal inflammation or fungal growth. The control of corneal neovascularization by VEGF-blockade is a promising adjunctive strategy in the management of microbial keratitis, and further studies should explore the safety and efficacy of topical antiangiogenic agents in keratomycosis.

In summary, corneal neovascularization occurs soon after the onset of corneal infection by *C. albicans*. Angiogenesis complicating fungal keratitis likely results from production of VEGF-A and other mediators such as MMP-9 that increase during corneal infection and inflammation. Inhibiting the activity of VEGF-A by a specific blocking antibody results in reduced corneal neovascularization without any apparent or unfavorable effects on innate immunity and fungal load. This study identifies a specific target for adjunctive chemotherapy aimed at reducing the sight-limiting consequences of microbial keratitis.
